# Suspected association of ventricular arrhythmia with air pollution in a motorbike rider: a case report

**DOI:** 10.1186/1752-1947-2-192

**Published:** 2008-06-03

**Authors:** Kent Emilsson

**Affiliations:** 1 Department of Clinical Physiology, Örebro University Hospital, SE-701 85 Örebro, Sweden

## Abstract

**Introduction:**

Premature ventricular complexes are to some extent a normal finding in healthy individuals and the prevalence increases with age and is more common in men. Premature ventricular complexes can occur in association with a variety of stimuli, and a lesser known cause is the association between air pollution and ventricular arrhythmias.

**Case presentation:**

A previously healthy man started to ride a lightweight motorbike in heavy traffic. A few weeks later he was admitted to hospital with premature ventricular complexes in bigeminy, which decreased after a few days when he was not exposed to exhaust fumes. A few weeks later he started using the motorbike again and the same symptoms developed once more, only to subside when he stopped riding in heavy traffic.

**Conclusion:**

Studies have shown an association between air pollution and premature ventricular complexes and other kinds of arrhythmias. The mechanism may be changes in cardiac autonomic function, including heart rate and heart rate variability. Air pollution should be considered when patients present with arrhythmias and no other causes are found.

## Introduction

To some extent premature ventricular complexes (PVCs) are a normal finding in healthy individuals and the prevalence increases with age and is more common in men. However, PVCs can occur in association with a variety of stimuli and can be produced by direct mechanical, electrical and chemical stimulation of the myocardium. Often they are noted in patients with false tendons, ischaemic or inflamed myocardium and during infection, hypoxia, anaesthesia or surgery. They can be provoked by a variety of medications, by electrolyte imbalance, by tension states, by myocardial stretch and by excessive use of tobacco, caffeine or alcohol [1]. A cause of PVCs that is not so well known among physicians is the association between air pollution and ventricular arrhythmias.

## Case presentation

A 43-year-old previously healthy man, a physician, living in a city of about 150,000 inhabitants, began to use a lightweight motorbike to commute to work, a distance of about 10 km, in heavy traffic in the middle of August 2006. He had previously been travelling in a car fitted with an air pollution filter, and had experienced no previous heart symptoms. He also walked or jogged about 6 km four to five times a week with no problems and was not on any medication. He felt relaxed and did not experience stress while riding the motorbike in heavy traffic. There were numerous traffic lights on his journey to work, which meant that he was forced to stop behind buses and trucks on several occasions where he experienced a strong smell of exhaust fumes.

After commuting to and from work by motorbike for about 2 weeks he began experiencing cardiac extrasystoles, something not previously experienced; on one occasion he was unable to sleep due to palpitations. He sought help and had an electrocardiogram (ECG) the following morning, which showed PVCs in bigeminy. The patient had also sinus tachycardia with a heart rate of about 110 beats per minute.

The patient was admitted to the cardiac intensive care unit and was examined using echocardiography, which was found to be normal, and there were no signs of false tendons. No ischaemia was seen on ECG and there were no signs of infarction. The frequency of PVCs began to decrease about 8 hours after admission. Blood tests showed no indications of infarction or infection, his blood glucose was normal and his lipid status and thyroid status were within normal limits. The patient had no fever. During the night and the next morning only a few PVCs and some premature atrial complexes were observed and the patient was discharged home. The diagnosis was given as myocarditis, although this diagnosis was uncertain.

The patient rested for 2 weeks with no further symptoms before returning to work. For the first few weeks he drove his car to work, but then began to use his motorbike again. Having used it for a few weeks on the same route he again began to experience extrasystoles and therefore contacted his physician, who recommended an exercise test and Holter ECG.

In the few days before the Holter ECG was applied the patient refrained from using his motorbike and began to feel better. Only a few PVCs and premature atrial complexes were found during 24 hours of Holter monitoring. The heart rate variability (HRV) showed a pattern with a somewhat high low-frequency to high-frequency ratio. An exercise test was carried out and the patient performed well, with no chest pain, arrhythmias or signs of ischaemia.

The patient began to believe that there was an association between using his motorbike and his symptoms and decided to stop using it. Since then no symptoms, apart from an occasional single extrasystole, have been noted by the patient.

## Discussion

In the present case there was no obvious explanation for the PVCs. Myocarditis was thought to be the underlying cause, even if this diagnosis was uncertain. This is a condition with various clinical presentations, from non-specific symptoms (fever, myalgias, palpitations or exertional dyspnoea) to fulminant haemodynamic collapse and sudden death. Myocarditis is difficult to diagnose and endomyocardial biopsy remains unequivocally the gold standard for establishing the diagnosis [2], although this cannot always be used in clinical practice. Cardiac biomarkers, especially troponin T or I, are now routinely measured by most clinicians when a clinical diagnosis of myocarditis is considered [2]. In the present case, however, the cardiac biomarkers creatine kinase and troponin T were not elevated.

Echocardiography can also help in the diagnosis of myocarditis, often showing left ventricular dysfunction and segment wall abnormalities in patients with biopsy-proven myocarditis [2]; here, however, the echocardiography was also normal. The patient had no obvious signs of ongoing infection and he had normal laboratory findings. Thus, myocarditis was an uncertain diagnosis in this case.

There was no electrolyte imbalance and the thyroid status was normal. There were no signs of ischaemia during the exercise test. The patient was a non-smoker and did not drink coffee or use alcohol. Thus, there was no obvious cause for the PVCs in this case.

A less well-known cause of PVCs is exposure to exhaust fumes from vehicles. Some studies have shown increased premature atrial complexes and PVCs in patients with implanted cardioverter defibrillators when exposed to high concentrations of air pollutants [3,4]. In addition to reported associations between PVCs and air pollution, there are also studies showing the association between air pollution and atrial fibrillation [5] and supraventricular extrasystoles and supraventricular tachycardia [6].

Most of the studies so far concerning arrhythmia and air pollution are on subjects with known heart disease; however, one study of healthy young non-smoking male highway patrol troopers in the United States has shown an increased number of premature supraventricular beats and changes in HRV [7].

The mechanism by which air pollution leads to cardiac morbidity and mortality remains unknown. Hypoxia has been suggested as a possible cause of air pollution-induced cardiac damage or vulnerability, but this has been shown not to be a culprit mechanism [8]. Pope et al. [9] suggest that it is due to changes in cardiac autonomic function, reflected by changes in mean heart rate and HRV. The inhaled environmental particles may promote a systemic sympathetic stress response, causing the heart rate to increase, the HRV to decrease and the ratio of low-frequency to high-frequency to be higher (as in the present case), causing ventricular tachyarrhythmias and ventricular fibrillation [8]. In a similar manner the inhaled particles also stimulate irritant receptors in the lung parenchyma and respiratory airways, which can lead to increased bronchoconstriction, increased vagal responses of the heart, increased HRV and increased high-frequency domain, possibly leading to bradyarrhythmias in appropriate settings [8].

A link has been demonstrated between carbon monoxide from vehicles and ventricular arrhythmias [3,10], but there are also studies that have shown that carbon monoxide has no significant effect on ventricular electrical stability or the frequency of ventricular ectopic activity [11,12].

In the present case it would have been of interest to obtain an ambulatory ECG during a motorbike ride in order to report changes in autonomic factors or changes in cardiac electrical instability in the myocardial substrate (as measured by T-wave alternans). This could not be done in the present case but could perhaps be performed in the future in a similar case. Moreover, while the subject did not report feeling stressed one cannot exclude the possibility that noise and stress contributed to the findings, and measures of cortisol, for instance using salivary cortisol, would have settled this issue. This was not possible in this case but may also be performed in the future if a similar case is noted.

## Conclusion

Studies have shown an association between air pollution and PVCs and other kinds of arrhythmias. The mechanism may be changes in cardiac autonomic function, including heart rate and HRV. Air pollution should be considered when patients present with arrhythmias and no other causes are found.

## Abbreviations

ECG; electrocardiogram; HRV: heart rate variability; PVC: premature ventricular complex.

## Competing interests

The author declares that they have no competing interests.

## Consent

Written informed consent was obtained from the patient for publication of this case report and any accompanying images. A copy of the written consent is available for review by the Editor-in-Chief of this journal.

## References

[B1] Olgin JE Zipes DP Zipes DP, Libby P, Bonow RO, Braunwald E Specific arrhythmias: diagnosis and treatment Braunwald's Heart Disease: A Textbook of Cardiovascular Medicine 2005 7 Philadelphia, PA: Elsevier Saunders 840 841

[B2] Magnani JW Dec GW Myocarditis: current trends in diagnosis and treatment Circulation 2006 113 876 890 10.1161/CIRCULATIONAHA.105.584532 16476862

[B3] Dockery DW Luttmann-Gibson H Rich DQ Link MS Schwartz JD Gold DR Koutrakis P Verrier RL Mittleman MA Particulate air pollution and nonfatal cardiac events. Part II. Association of air pollution with confirmed arrhythmias recorded by implanted defibrillators Res Rep Health Eff Inst 2005 124 83 126 17153518

[B4] Rich DQ Schwartz J Mittleman MA Link M Luttmann-Gibson H Catalano PJ Speizer FE Dockery DW Association of short-term ambient air pollution concentrations and ventricular arrhythmias Am J Epidemiol 2005 161 1123 1132 10.1093/aje/kwi143 15937021

[B5] Rich DQ Mittleman MA Link MS Schwartz J Luttmann-Gibson H Catalano PJ Speizer FE Gold DR Dockery DW Increased risk of paroxysmal atrial fibrillation episodes associated with acute increases in ambient air pollution Environ Health Perspect 2006 114 120 123 16393668 10.1289/ehp.8371 PMC1332666

[B6] Berger A Zareba W Schneider A Rückerl R Ibald-Mulli A Cyrys J Wichmann HE Peters A Runs of ventricular and supraventricular tachycardia triggered by air pollution in patients with coronary heart disease J Occup Environ Med 2006 48 1149 1158 10.1097/01.jom.0000245921.15916.03 17099451

[B7] Riediker M Devlin RB Griggs TR Herbst MC Bromberg PA Williams RW Cascio WE Cardiovascular effects in patrol officers are associated with fine particulate matter from brake wear and engine emissions Part Fibre Toxicol 2004 1 2 10.1186/1743-8977-1-2 15813985 PMC1074349

[B8] Stoen PH Godleski JJ First steps toward understanding the pathophysiologic link between air pollution and cardiac mortality Am Heart J 1999 138 804 807 10.1016/S0002-8703(99)70002-5 10539808

[B9] Pope CA III Verrier RL Lovett EG Larson AC Raizenne ME Kanner RE Schwartz J Villegas M Gold DR Dockery DW Heart rate variability associated with particulate air pollution Am Heart J 1999 138 890 899 10.1016/S0002-8703(99)70014-1 10539820

[B10] Dockery DW Luttmann-Gibson H Rich DQ Link MS Mittleman MA Gold DR Koutrakis P Schwartz JD Verrier RL Association of air pollution with increased incidence of ventricular tachyarrhythmias recorded by implanted cardioverter defibrillators Environ Health Perspect 2005 113 670 674 15929887 10.1289/ehp.7767 PMC1257589

[B11] Verrier RL Mills AK Skornik WA Acute effects of carbon monoxide on cardiac electrical stability Res Rep Health Eff Inst 1990 35 1 14 2291816

[B12] Dahms TE Younis LT Wiens RD Zarnegar S Byers SL Chaitman BR Effects of carbon monoxide exposure in patients with documented cardiac arrhythmias J Am Coll Cardiol 1993 21 442 450 7678844 10.1016/0735-1097(93)90687-v

